# Performance of Anaerobic Digestion of Chicken Manure Under Gradually Elevated Organic Loading Rates

**DOI:** 10.3390/ijerph16122239

**Published:** 2019-06-25

**Authors:** Fei Wang, Mengfu Pei, Ling Qiu, Yiqing Yao, Congguang Zhang, Hong Qiang

**Affiliations:** 1 College of Mechanical and Electronic Engineering, Northwest A&F University, Yangling 712100, China; alwaysinholiday@outlook.com (F.W.); jessehan1994@gmail.com (M.P.); dzhtyao@126.com (Y.Y.); 2 Western Scientific Observation and Experiment Station of Development and Utilization of Rural Renewable Energy of Ministry of Agriculture, Northwest A&F University, Yangling 712100, China; 3 College of Resources and Environment, Northwest A&F University, Yangling 712100, China

**Keywords:** anaerobic digestion, chicken manure, organic loading rates, ammonia inhibition, microbial biomass yield

## Abstract

Poultry manure is the main source of agricultural and rural non-point source pollution, and its effective disposal through anaerobic digestion (AD) is of great significance; meanwhile, the high nitrogen content of chicken manure makes it a typical feedstock for anaerobic digestion. The performance of chicken-manure-based AD at gradient organic loading rates (OLRs) in a continuous stirred tank reactor (CSTR) was investigated herein. The whole AD process was divided into five stages according to different OLRs, and it lasted for 150 days. The results showed that the biogas yield increased with increasing OLR, which was based on the volatile solids (VS), before reaching up to 11.5 g VS/(L·d), while the methane content was kept relatively stable and maintained at approximately 60%. However, when the VS was further increased to 11.5 g VS/(L·d), the total ammonia nitrogen (TAN), pH, and alkalinity (CaCO_3_) rose to 2560 mg·L^−1^, 8.2, and 15,000 mg·L^−1^, respectively, while the volumetric biogas production rate (VBPR), methane content, and VS removal efficiency decreased to 0.30 L·(L·d)^−1^, 45%, and 40%, respectively. Therefore, the AD performance immediately deteriorated and ammonia inhibition occurred. Further analysis demonstrated that the microbial biomass yield and concentrations dropped dramatically in this period. These results indicated that the AD stayed steady when the OLR was lower than 11.5 g VS/(L·d); this also provides valuable information for improving the efficiency and stability of AD of a nitrogen-rich substrate.

## 1. Introduction

It is estimated that a total quantity of 4 × 10^8^ t/year of chicken manure is being produced in China, and most of it will be transferred into the natural environment directly, causing serious environmental problems [1]. Using energy conversion technologies such as anaerobic digestion (AD) to deal with various bio-wastes like chicken manure, cow dung, and other various agricultural residues has been an efficient and popular approach for many years because it produces clean energy without polluting the environment [2,3]. However, it is common for ammonia inhibition to have a negative impact on the stability of AD systems [4] and to even cause AD failure when the ammonia concentration increases to a certain range [5,6]. Ammonia inhibition, especially that caused by free ammonia (NH_3_), can change the intracellular pH and make key enzymes inactive through penetrating into microbial cells and interfering with normal bio-chemical reactions [7]. Ammonia inhibition in the AD treatment of chicken manure is mainly due to free ammonia nitrogen (FAN), which can be converted from protein and uric acid during the AD process [8] and is even more toxic than NH^+^ under artificial adding conditions [7].

In previous studies that are devoted to reducing the ammonia inhibition of an AD system, the TS (total solids) of the substrate was proven to be a critical parameter in influencing the comprehensive performance and working efficiency of the AD system [9]. Although reducing the TS means less ammonia input, it consumes more energy and water resources. Co-digestion of different materials may be a favorable way to prevent inhibition while significantly increasing the organic loading rate (OLR), but it needs certain amounts of special kinds of feedstocks with various chemical compositions [10]. In order to increase the TS concentration and improve the AD performance, co-digestion of nitrogen-rich materials and carbon-rich materials in an appropriate ratio could relieve the total ammonia nitrogen (TAN) inhibition to some extent [11]. A high TS can reduce the required volume of reactors, but it will be easier for the reactors to accumulate ammonia. Therefore, a substrate with an appropriate TS can be regarded as a significant factor for AD performance. According to Dalkılıc et al., AD performance began to deteriorate when the TS reached 8.25% and the TAN concentration reached 3000 mg·L^−1^. However, it was once reported that the AD process was sustained for a certain period under the condition of 11.2% TS when the TAN concentration reached 5000 mg·L^−1^ [12]. Besides this, there is also a dispute regarding AD inhibition at different levels of TAN concentrations. Wu et al. found that the methane content was not steady once the TAN concentration was over 3000 mg·L^−1^, while other scholars had pointed out that the AD process would be inhibited under both mesophilic and thermophilic inoculation conditions once the TAN concentration exceeded 2500 mg·L^−1^ [13]. It was even observed that some certain methanogens were inhibited when the TAN concentration reached 1700 mg·L^−1^ [14]. According to Astals (2018), TAN may join with FAN to cause inhibition [15]. Because the existence of TAN inhibition interacts with a series of factors including pH and temperature, which eventually change the FAN level to some extent, different conditions can uncertainly influence the ultimate TAN limitation.

As a result, many researchers are dedicated to finding relatively accurate limitations of TAN and FAN concentrations which can inhibit the AD process. Although some studies concerning the relationship between OLR and AD performance have been reported [16], and the effects of the OLR of nitrogen-rich substrates on TAN cumulation were also investigated under specific OLR concentrations [17], there is also an urge to explore the more complex relationship between TAN, FAN, and OLRs, especially with an innovative feeding means. As is known to us, as a raw substrate with high nitrogen content, chicken manure easily causes TAN inhibition during AD [18]. It is of great significance to explore the accumulation levels of TAN and FAN under different OLRs, as well as their influence on the general AD performance of chicken manure, which is essential for the future treatment of high-nitrogen substrates like chicken manure. To investigate the above issues, it is necessary to examine the impacts of different TAN concentrations on the AD performance of chicken manure; therefore, the AD performance needs to be evaluated with the help of a series of representative indices as well. 

In addition, kinetic analysis is a relatively precise method used to evaluate and predict the performance of AD in various situations [19,20,21]. Zhang et al. investigated the AD of food waste under different OLRs and described the kinetic performance by several models. Guo et al. examined the microbial biomass yield during the AD process through employing the substrate mass balance model, in which the microbial biomass content was measured in terms of volatile suspended solids (VSS) [19]. Although modelling the microbial biomass balance under different OLR conditions has been studied, few investigations have concentrated on the relationship between different VS concentrations and microbial biomass yields throughout the AD, especially under the effect of TAN accumulation. Therefore, it is necessary to evaluate the microbial biomass concentration tendency in an AD reactor fed with a nitrogen-rich substrate.

Based on the above, five gradient OLRs of diluted chicken manure from 3.5 g VS/(L·d) to 15 g VS/(L·d) (3.5, 5.5, 8, 11.5, 15 OLR) were set up in a continuous stirred tank reactor (CSTR), with a hydraulic retention time lasting 30 days. Because the CSTR has a more stable environment for microbial processes compared with batch reactors, it is possible to increase the acclimations of these microbes. Hence, the aims of this study are to (1) investigate the formation trend of ammonia inhibition inside the AD system with gradient OLRs, (2) analyze the detailed AD performance at different ammonia concentrations to get a clearer picture of the inhibition mechanism, (3) study the relationship between feeding VS concentrations and microbial biomass concentration under the effect of accumulative TAN using microbial biomass yield models, and (4) eventually identify the suitable OLR for AD of chicken manure.

## 2. Materials and Methods 

### 2.1. Chicken Manure and Inoculum

Chicken manure were obtained from the Third Farm of Northwest A&F University, Shaanxi, China. After removing the debris, the chicken manure was crushed and mixed into a homogenized matrix using a blender and then stored in a refrigerator at 4 °C before use. Activated sludge obtained from a local thermophilic AD system was used as an inoculum and was collected from the Fifth Sewage Treatment Plant in Xi’an, Shaanxi. The physico-chemical characteristics of the chicken manure and inoculum are summarized in Table 1.

### 2.2. Experimental Setup

**Figure 1:**
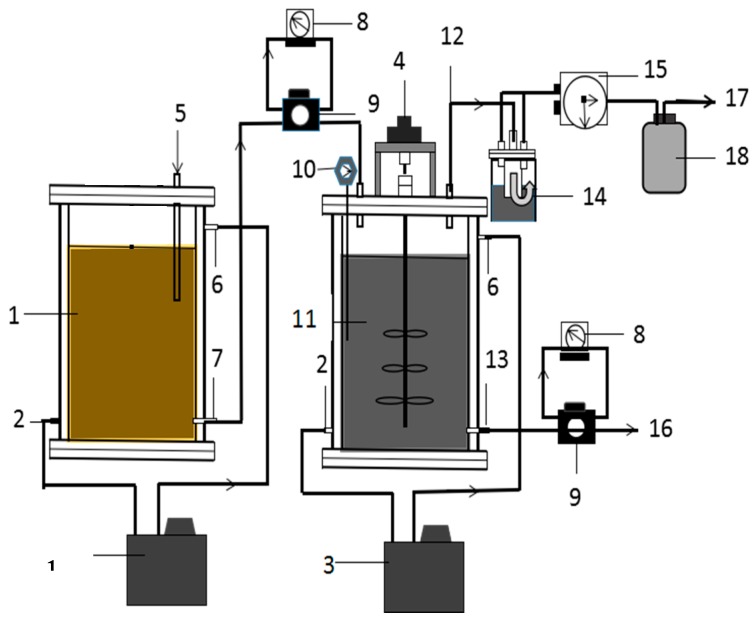
Scheme of the design of the present continuous stirred tank reactor (CSTR). 1. Substrate tank; 2. Circulation effluent port; 3. Warming circulation groove; 4. Electromagnetism stirring device; 5. Substrate addition port; 6. Circulation influent port; 7. Substrate effluent port; 8. Time controller; 9. Peristaltic pump; 10. Thermometer; 11. Reactor; 12. Biogas outlet; 13. Effluent port; 14. Water and gas separator; 15. Wet biogas meter; 16. Effluent; 17. Biogas export; 18. Desulfurization bottle; 19. Cooling water circulation groove.

The experimental CSTR setup is shown in Figure 1. In this study, the total volume of the anaerobic reactor was 8 L, including an effective working volume of 6 L. The temperature was adjusted by a water jacket surrounding the reactor so that the digestion chamber could be kept at a constant mesophilic temperature of 35 ± 0.5 °C. Two duplicate reactors were used in this experiment to minimize the chance error. All the reactors were sealed by caps with a stirrer in the middle and an outlet that connected to a biogas bag. All the biogas generated by the CSTR was quantified through a wet gas flowmeter before proceeding to these bags. Besides this, the temperature of the substrate tank was set at 4 °C to prevent the biological decomposition of the raw material before its use, as the experiment lasted nearly half a year. To keep the raw material and inoculum well distributed, the mechanical stirring device of the reactor continued stirring at a rotating speed of 60 rpm during the whole process of the experiment.

### 2.3. Experimental Design

To all these reactors were added 450 g chicken manure and 1500 g inoculum sludge based on the wet weight. Deionized water was added to meet the required VS concentration (2.1%). Then, the reactor began to operate with a hydraulic retention time of 30 days and an OLR of 3.5 g VS/(L·d). The methane production remained stable for 2~3 HRTs (hydraulic retention time); after that, diluted chicken manure was added as the sole substrate at the gradient loading levels, and the OLRs were increased from 3.5 to 15.0 g VS/(L·d) (see Table 2). To keep a constant volume for these reactors, all the reactors were drained of a quantity equivalent to that of the substrate that was added to the reactors. The precise quantity of diluted chicken manure from the substrate tank was pumped into the reactor automatically every day through a peristaltic pump in which a time procedure was embedded (4, 8, 12, 16, 20, and 24 o’clock each day). Therefore, a total amount of 204 mL of diluted substrate was divided into six equivalent parts to inject into the reactor, which means a quantity of 34 mL of substrate was added for each part. In other words, each group of substrates sent to the reactor was no more than 1% of the workings volume of the anaerobic reactor in order to minimize the influence of feeding on the AD system and therefore keep the whole AD process stable. The whole experiment was divided into five stages lasting 150 days based on different organic loadings (Stages 1~5, ranging from 3.5 to 15.0 g VS/(L·d)), with each stage beginning to operate when the biogas production in the previous stage became stable. 

In order to analyze the characteristics of samples, liquid samples were collected twice a week, and a biogas sample was analyzed every day. For statistical reasons, triplication was set for each condition. The detailed characteristics of the digestate inside the anaerobic reactor at different stages are shown in Table 2.

### 2.4. Analytical Methods

The pH value was measured by using a digital pH meter (PB-10, sartorius, China) for this experiment. TCOD (total chemical oxygen demand) was measured by the rapid digestion colorimetry method [22]. The liquid samples were treated by a high-speed refrigerated centrifuge (HC-3018R, Zonkia Scientific Instruments Co. Ltd., China) at 4 °C and 15,000 rpm for 20 min. The total alkalinity, TAN concentrations, soluble chemical oxygen demand (SCOD), TS, and VS were all determined by the standard methods of the American Public Health Association [23]. Free ammonia nitrogen (FAN) can be calculated via following Formula (1) [12]:

(1) $$\frac{FAN}{TAN}={\left[1+\frac{10^{-pH}}{10^{-(0.9018+\frac{2729.92}{T(K)})}}\right]}^{-1}$$

where FAN and TAN are the concentrations of free ammonia nitrogen and total ammonia nitrogen, respectively, in mg/L; the pH value was measured from the liquid phase at fixed temperature T (°K).

The daily biogas yield was measured by a wet gas flowmeter (W-NK-0.5A, SINAGAWA, Japan), the composition of the biogas was analyzed by gas chromatography (GC-2014, Shimadzu, Japan) facilitated by a thermal conductivity detector (TCD) (heater 150 °C, helium flow: 10 mL/min), and the temperatures of the gas entrance and column oven were 100 °C and 80 °C, respectively. Volatile fatty acids (VFAs) were detected using gas chromatography (GC-2014, Shimadzu, Japan) facilitated by a flame ionization detector (FID) (heater 250 °C, nitrogen flow: 30 mL/min). The size of the chromatographic column was Φ 30 m × 0.82 mm (stablilwax-DA Column). The temperature of the column oven was set in an automatic heating procedure which began heating at 80 °C with a ramp rate of 10 °C/min, reached 150 °C, and held this for 6 min.

### 2.5. Statistical Method

Pearson correlation analysis was applied in the study to determine whether the parameters observed in the experiment were significant or not. The *p* values were calculated using IBM SPSS Statistics 19.0 (IBM Inc., Armonk, NY, USA) and were compared with cutoff values of 0.05 and 0.01.

## 3. Results and Discussion

### 3.1. Effect of OLRs on AD Performance

#### 3.1.1. Effect of OLRs on Methane Production

**Figure 2:**
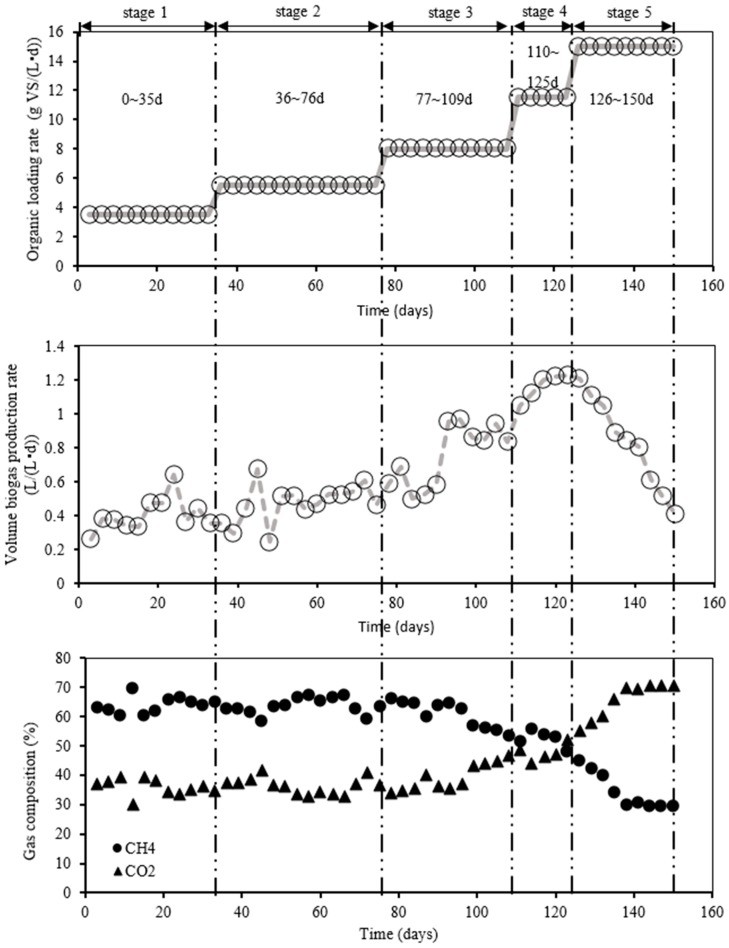
Effect of OLR on the volumetric biogas production rate and gas composition.

The experiment was conducted for 150 days in total and was divided into five stages (Stages 1–5) according to the different OLRs. The fluctuations in the volumetric biogas production rate (VBPR) and the biogas composition (i.e., methane and CO_2_ contents) along with the AD reactor operation time are shown in Figure 2. Overall, in the first four stages, the VBPR gradually increased with the increase of the influent OLR; although in the fourth stage the methane content was slightly lower than in the first three stages, the total yield of methane still increased. Therefore, these four stages could be regarded as a stable period from the methane yield perspective. Then, both the VBPR and methane content decreased suddenly during the fifth stage. The methane content remained stable (around 65%) during the first three stages and was maintained at around 55% in the fourth stage but sharply dropped to 25% during the last stage. Consequently, the fourth stage was a transition stage, because the methane content in this stage diminished but still maintained relatively stable; since the VBPR was still increasing, the total volume of methane even improved slightly.

From Day 1 to Day 35 (Stage 1), the influent OLR was 3.5 g VS/(L·d), and the VBPR increased from 0.31 L/(L·d) to 0.44 L/(L·d). In this period, the methane content was maintained at about 60%, indicating that the AD was running quite stably. Unlike batch experiments where the carbon dioxide content was greater than the methane content [24], this CSTR reactor had a successful startup. Then, from Day 36 to Day 76 (Stage 2), the influent OLR was 5.5 g VS/(L·d). Compared with Stage 1, the methane content was almost the same, while the VBPR was a little bit higher than that in Stage 1. However, the methane content in Stage 3 (from Day 77 to Day 109) was lower than in the previous stages and finally stabilized at around 55%. Although the OLR in this stage was up to 8 g VS/(L·d), the VBPR still increased and stabilized at 0.75 L/(L·d). From Day 110 to Day 125 (Stage 4), the OLR was increased to 11.5 g VS/(L·d); the VBPR rapidly rose to around 1.20 L/(L·d) and remained stable, while the methane content stayed above 50%. According to Roubík [25], the methane content in stable AD which used animal manure as the main feedstock was almost over 60%. Hence, the methane content in the fourth stage was already unstable. In the last stage (from Day 126 to Day 150, with OLR of 15 g VS/(L·d)), there was a sharp decline in both VBPR and methane content, decreasing to 0.41 L/(L·d) and 30%, respectively. Therefore, taking the VBPR and methane content into consideration, the proposed value of OLR ought to be no more than 11.5 g VS/(L·d), under which the anaerobic reactor can run smoothly, or even less to ensure a stable methane content.

#### 3.1.2. Intermediate Products in the AD Reactor

**Figure 3:**
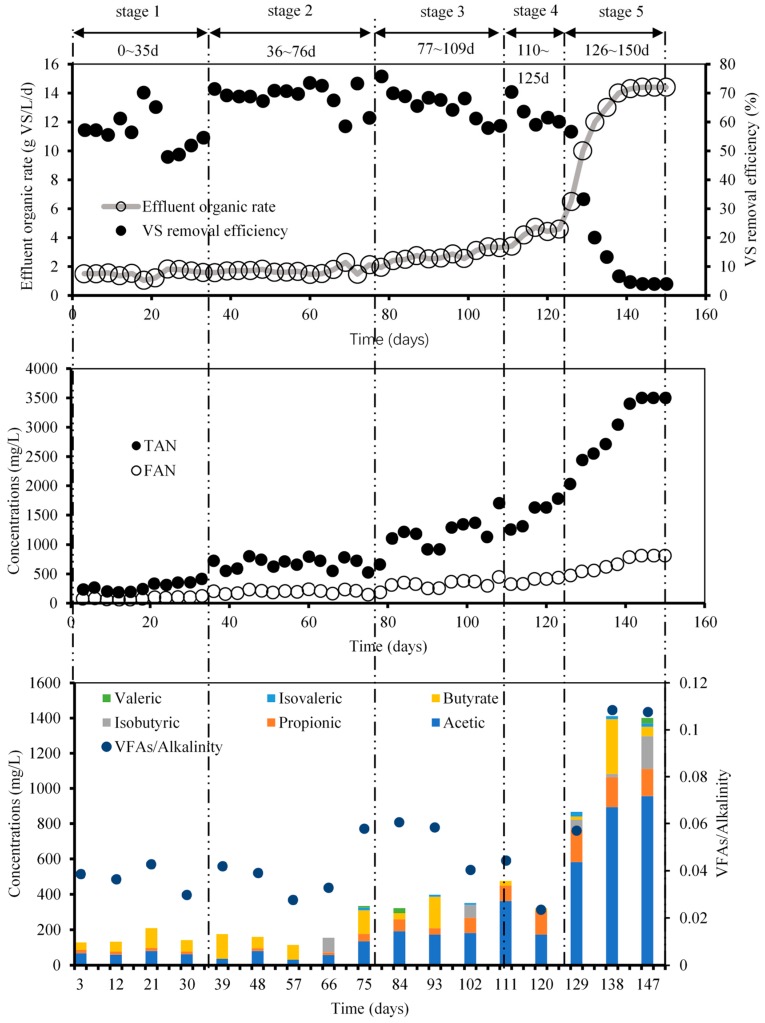
Effect of OLR on the VS removal efficiency, effluent organic rate, and the TAN, free ammonia nitrogen (FAN), and volatile fatty acid (VFA) concentrations.

To investigate the AD performance of chicken manure at different OLRs, the changes in the effluent VS concentrations, VS removal efficiency, and TAN and FAN concentrations with proceeding AD are depicted in Figure 3. When the OLR was at a relatively low concentration, the effluent organic rate ranged from 2 to 4 g VS/(L·d) during the first three stages, indicating that AD was quite stable with a considerably high VS removal efficiency. Although the VS removal efficiency was stable in the fourth stage, the methane content declined gradually, indicating that the reactor began to deteriorate. At the beginning of the fifth stage, there was a steep climb in the effluent organic rate, and the value surged up to 14 g VS/(L·d), while the OLR at this stage was 15 g VS/(L·d). Meanwhile, the VS removal efficiency of the process showed the opposite tendency: a sharp drop appeared in the last stage, indicating that the reactor failed to operate normally under such a relatively high OLR condition. On the other hand, the TAN concentration accumulated bit by bit in the first four stages, in which it was always below 2000 mg/L, but rose up to 3500 mg/L immediately when the OLR reached 15.5 g VS/(L·d). The FAN concentration increased from 61 mg/L to 558 mg/L during the AD process. 

It can be seen in Figure 3 that inhibition was not found from Stage 1 to Stage 4, when the TAN and FAN concentrations were under 1600 mg/L and 500 mg/L, respectively, indicating that the AD system was not inhibited when the organic loading OLRs were no more than 11.5 g VS/(L·d). Additionally, the ever-increasing TAN in the fourth stage should be noted as it shows that inhibition had already accumulated at that time. However, when the OLR reached 15 g VS/(L·d), the TAN and FAN concentrations reached 2560 mg/L and 558 mg/L, respectively, which eventually caused the failure of the AD.

Figure 3 also illustrates that the tendencies of FAN and TAN both changed substantially in the last two stages; although the absolute rise of TAN was higher than that of FAN, the ammonia inhibition still had a strong connection with both TAN and FAN. Dalkılıc et al. reported that when the TAN concentration reaches 3000 mg/L, failure of AD will occur [9]. The OLR in Stage 5 was 15 g VS/(L·d) and the TAN concentration reached nearly 3500 mg/L, which was almost in accordance with the previous results obtained in this study. As a result, the feeding process was ceased on Day 150 to prevent any deterioration of the reactor. Additionally, as was reported previously, the VFAs increase along with the accumulation of TAN, which causes serious inhibition in AD systems [5,26]. However, in Stage 5 of this experiment, the VFAs reached a highest value of 1412 mg/L, which was much lower than the threshold value (2500 mg/L) of the inhibition reported previously [27]. The levels of some of the VFAs did not keep increasing; in other words, they declined during a certain period, like iso-butyric acid (declined from Day 120 to Day 140). However, as can be seen from Figure 3, the highest proportions of VFAs-acetic acid, showed an growth trend during the whole experiment. Among the VFAs, that with the largest proportion among the six main components of VFAs was acetic acid, which reached its maximum (894.29 mg/L) in the last period of the experiment. In addition, propionic, butyrate, and iso-butyrate acid all cumulated in the fifth stage, indicating that the bioconversion and microbial activity were relatively weaker under high ammonia concentrations [28]. Owing to the high TAN in this stage, a huge amount of VFA accumulation could be attributed to the rising TAN and FAN concentrations. This may be attributed to the valuable acetogenic communities because they are inhibited seriously by high ammonia levels [29]. According to Yang et al., high ammonia suppresses the acetolactic pathway; that is why acetic acid accumulated hugely in the fifth stage [30]. Similarly, the decreases in the methane content and VBPR in the fifth stage may also be due to the ever-increasing TAN and VFA concentrations.

#### 3.1.3. Organic Removal Efficiency of the AD System

**Figure 4:**
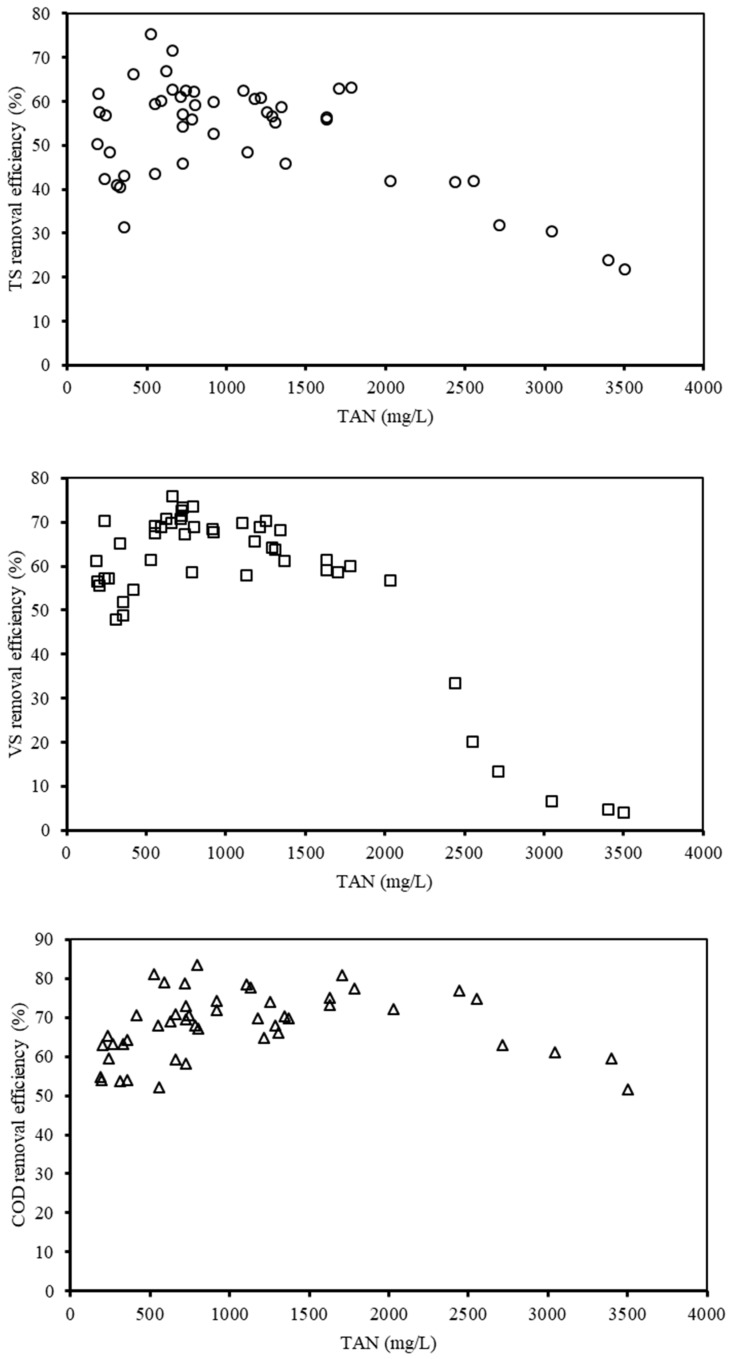
Effect of TAN on different organic removal efficiencies. (TAN is not an independent variable here; it may also correspond to one or more dependent values.).

The aim of applying the AD technique is mainly to convert the organic parts of biowastes into biogas; therefore, the removal efficiency of organic wastes was adopted as the main indicator for evaluating the AD performance. In this study, the AD efficiency was mainly assessed by the TS, VS, and COD removal efficiencies. However, the AD efficiency was also influenced by the TAN. Hence, all these factors should be considered to reveal the mechanisms of ammonia inhibition. In Figure 4, the variation trends of the TS, VS, and COD removal efficiencies with respect to the TAN concentrations are depicted and analyzed in detail. The TS and VS removal efficiencies showed the same tendency before the TAN concentration reached 1600 mg/L. After that, along with the increasing TAN concentration, both the TS removal efficiency and VS removal efficiency declined constantly to 20% and 10%, respectively, as the TAN concentration rose up to 3500 mg/L. However, the COD removal efficiency reached around 50% with increasing TAN concentration. From the Pearson correlation analysis, it was found that both the TS removal efficiency and VS removal efficiency exhibited a negative relationship with TAN concentration (*p* < 0.05), while the COD removal efficiency revealed no significant relationship (*p* > 0.05). This phenomenon could be explained by the well-known knowledge that increasing OLR stimulates organic removal efficiency at a lower range of TAN concentration. According to previous studies [31], when the TAN concentration is under 800 mg/L, the anaerobic organisms in the AD system are under acclimation and finally adjust to the various anaerobic environments.

Afterwards, the organic removal efficiency (especially based on TS and VS) dropped as the TAN concentration continued to rise. Besides this, applying the indicator of COD removal efficiency can more precisely represent the overall removal efficiency of the AD system than TS. COD tended to increase when the anaerobic organisms were under the period of acclimation, while the TS remained almost unchanged. This finding supported the previous result that AD would be inhibited beyond a certain level of TAN concentration [32].

#### 3.1.4. Relationship between TAN Levels and AD Stability

**Figure 5:**
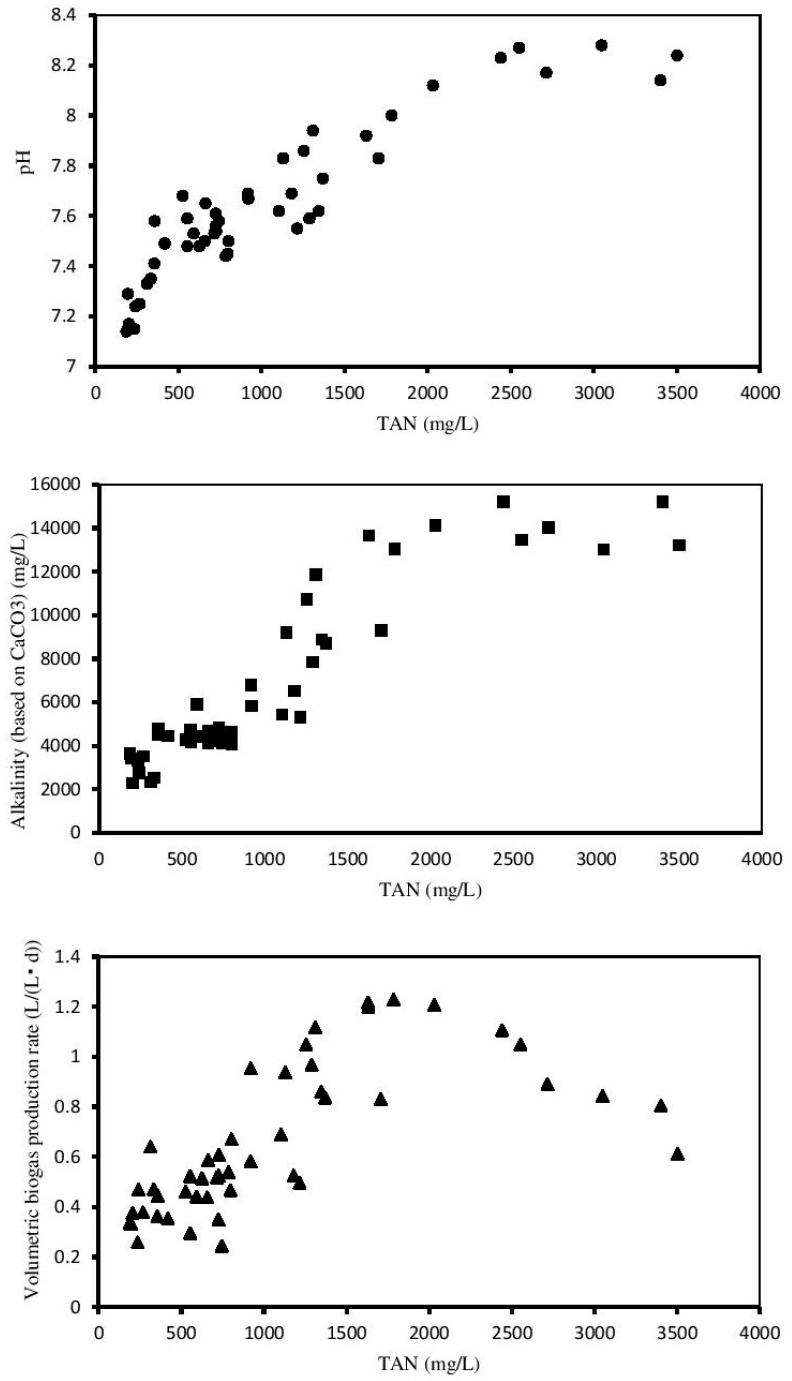
Effect of TAN on the pH, alkalinity, and volumetric biogas production rate. (TAN is not an independent variable here; it may also correspond to one or more dependent values.).

One of the most effective methods to evaluate the AD stability is to investigate the quantity of ultimate system products (e.g., CH_4_) and the intermediate metabolism products existing in liquid phase (pH and alkalinity) under different TAN concentrations. Overall, the pH and alkalinity continued to rise with increasing TAN concentration, while the VBPR rose to 1.2 L/(L·d) when the TAN concentration reached 1600 mg/L and then decreased gradually thereafter (Figure 5).

The pH value showed a very significantly positive relationship with TAN concentration (*p* < 0.01), with a continual increase from 7 to 8.2. It was reported in previous findings that the suitable pH value range for AD is 6.5 to 8.0 [33]. When the TAN concentration was under 1600 mg/L, the pH value for the AD reactor was almost below 8.0, indicating that excessive TAN concentration may result in an extremely high pH value; this was in agreement with a previous study [34]. In return, the increased pH would accelerate the release of ammonia; hence, the alkalinity rose along with the pH [35]. Similarly, the alkalinity (based on CaCO_3_), which reflects the buffer capacity of the AD system, showed the same tendency as the pH value. However, there was a very significantly positive relationship between alkalinity and TAN concentration (*p* < 0.01), indicating that high alkalinity emerged when the TAN concentration was beyond 1600 mg/L. However, the VFAs/Alkalinity ratio was gradually growing, reaching 0.1 in the fifth stage, indicating that the VFA concentration was rising faster than the total alkalinity (Figure 3). Although the ratio was below the value of 0.5 which was reported by Li [36] and was closer to the 0.24 and 0.19 calculated by Chiumenti [37], this result may be due to the different substrates used in the experiment. This is because lignocellulosic materials tend to produce more VFAs in anaerobic conditions than manure.

Although the VBPR exhibited a significant relationship with the TAN concentration (0.01 < *p* < 0.05) overall, it began to decline when the TAN concentration was over 1600 mg/L, which basically happened in the fifth stage. This outcome was also accordant with the results obtained before, which confirmed that the threshold of ammonia inhibition was approximately 1600 mg/L.

### 3.2. Effects of VS Concentration on Microbial Biomass Yield

#### 3.2.1. Substrate Mass Balance

The substrate mass balance equation was selected to evaluate the different kinds of organic degradation rates during the AD processes that have been reported before, such as those using food wastes [20], pig manure [38], and chicken manure [21]. According to the substrate mass balance equation and the performance of AD with the gradient OLRs of the substrate, the process could be given by Equations (2) and (3) based on VS:

(2) $$QS_{in}=QS_{out}+q_{biogas}\cdot Y_{S/B}$$

(3) $$QS_{in}=QS_{out}+q_{methane}\cdot Y_{S/B}$$

where Q is the flow rate (L/d); S_in_ is the VS concentration in the influent (g VS/L); S_out_ is the VS concentration in the effluent (g VS/L); Y_S/B_ is a coefficient representing the removed substrate transferred into biogas (g VS_removal_/L); Y_S/M_ is a coefficient representing the removed substrate transferred into methane (g VS_removal_/L); q_biogas_ is the daily biogas yield (L/d); and q_methane_ is the daily methane yield (L/d).

Regrouping the terms in Equations (2) and (3) gets two new equations as follows:

(4) $$Q(S_{in}-S_{out})/q_{biogas}=Y_{S/B}$$

(5) $$Q(S_{in}-S_{out})/q_{methane}=Y_{S/B}.$$

According to Equations (4) and (5), if Q(S_in_-S_out_) is plotted against q_biogas_ and q_methane_, the slopes of the obtained straight lines will be Y_S/B_ and Y_S/M_, respectively. 

**Figure 6:**
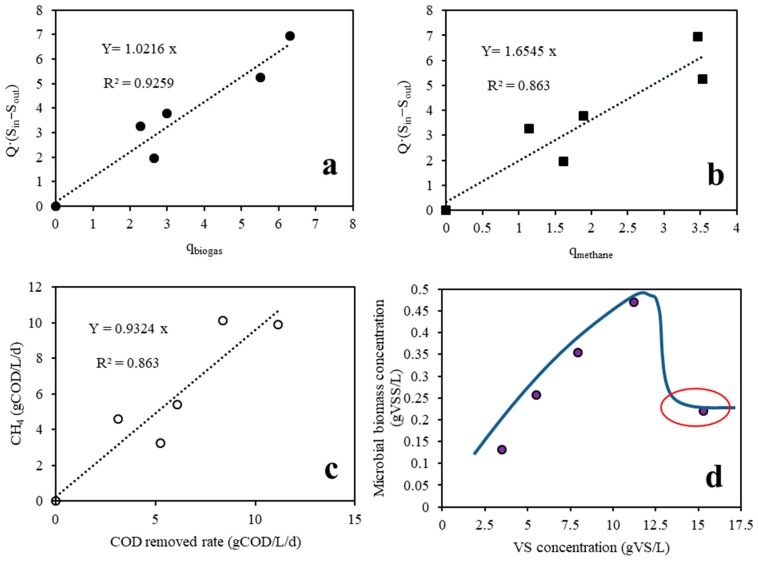
(**a**) Variation in Q·(S_in_ − S_out_) as a function of daily biogas production; (**b**) Variation in Q·(S_in_ − S_out_) as a function of daily methane production; (**c**) VMPR as a function of the volumetric COD removal efficiency; (**d**) Variation in the calculated microbial biomass concentration as a function of the VS concentration.

As can be seen from Figure 6a,b, Y_S/B_ and Y_S/M_ were 1.0216 g VS_removal_/L and 1.6545 g VS_removal_/L, respectively. Based on these slope coefficients, the biogas and methane yield coefficients were calculated as 0.9197 L/g VS_removal_ and 0.6044 L/g VS_removal_, respectively. These results were very similar to the results reported by Zhang et al., who found that the biogas and methane yield coefficients were 0.912L/g VS_removal_ and 0.560L/g VS_removal_, respectively, when the hydraulic retention time was varied from 20 to 52 days and using chicken manure as the sole substrate [21].

#### 3.2.2. Microbial Biomass Yield Values

At standard temperature and pressure (STP), to produce one mol of methane (22.4 L), 2 mol of oxygen-equivalent COD will be destroyed (64 g). In other words, 1 L methane production is equivalent to 2.86 g of COD destruction at STP. In this study, 1 g VS is equivalent to 1.27g COD, which is below the 1.68 g COD reported by Zhang et al. (2017). This model was used to evaluate the microbial biomass yield under different AD conditions, including continued feeding with different OLRs [20,38], or with different hydraulic retention times [20], or with different temperatures [19]. If the volumetric methane production rate (VMPR) is plotted against the volumetric COD removal efficiency, as can be seen from Figure 6c, the VMPR increases at a rate of 0.93 CH_4_-COD/g COD_removed_ with increasing COD removal. Therefore, around 6.76% of the removed COD will be converted into microbial biomass. It was assumed that the chemical structural formula of the microbial biomass was C_5_H_7_O_2_N, and the obtained microbial biomass yield was 1.41g COD/g VSS [39]. In this study, the microbial biomass yield (Y) was 0.0479 g VSS/g COD_removed_, which was almost in accordance with results reported by other researchers who used the same calculation method. Table 3 gives the ranges of these obtained microbial biomass yield values.

#### 3.2.3. Microbial Biomass Balance

The microbial biomass balance model for net microbial growth are given in Equation (6):

(6) $$\frac{dX}{dt}V=QX_{out}-QX_{in}+r_{g}^{\prime }V$$

where V is the effective volume of the digester (L), Q is the flow rate of the influent (L/d), X_out_ is the microbial concentration in the effluent (g/L), X_in_ is the microbial concentration in the influent (g/L), and $r_{g}^{\prime }$ is the net growth rate of the microbials (g/(L·d)).

According to the outcomes of Zhang et al. (2017), $r_{g}^{\prime }$ in Equation (6) should be expressed by Equation (7):

(7) $$r_{g}^{\prime }=\frac{\mu_{\max}SX_{out}}{K_{S}+S}-K_{d}\cdot X_{out}=\frac{YKSX}{K_{S}+S}-K_{d}$$

where μ_max_ is the maximum specific growth rate of the microbials (d^−1^), S is the concentration of the substrate (g/L), K_s_ is the half rate constant (g/L), and K_d_ is the microbials decay coefficient (d^−1^).

According to the theory reported by Guo [38], if the CSTR is in a stable state, then dX/dt equals 0. Besides this, due to the concentration of microbials in the influent of the reactor being very low, it could be negligible during the calculation. Based on these two principles, the microbial biomass concentration can be obtained by Equation (8):

(8) $$X=\frac{Y\cdot (S_{in}-S_{out})}{1+K_{d}\theta_{c}}$$

where θ_c_ is the hydraulic retention time of AD (days).

Based on Equation (8), the microbial biomass concentrations under different VS concentrations can be calculated using Y and K_d_ obtained above. The relationship between the microbial biomass concentration and the VS concentration is presented in Figure 6d. It can be seen that the microbial biomass concentration increased with increasing VS concentration and met its peak of 0.47 g VSS/L when the VS concentration reached 11.5g VS/L. This may support the conclusion that both the degradation ability and methane production will increase with rising VS concentration under a certain level, which is also in accordance with the outcomes reported by Guo [38]. Throughout the first four stages, microorganisms in the anaerobic reactor seemed to be acclimatized and endured a higher ammonia concentration. Although the calculated microbial biomass was still increasing during the fourth stage, the operation status was slightly unstable due to the decreasing methane content and cumulating ammonia inhibition. According to Chen et al., some microorganisms would open up alternative pathways to resist high ammonia in the environment, and the AD system may be able to recover finally [43]. When the VS concentration reached 15.5 g VS/L, the microbial biomass concentration dropped sharply to 0.22 g VSS/L and met its minimum. This result may be attributed to the excessive accumulative TAN in the anaerobic reactor, as TAN reached beyond the threshold value of TAN tolerance of microbials and thus affected and inhibited the metabolism of these microorganisms [44]. This may be the major reason as to why there was a sharp decline in the VBPR and methane content. Notably, the microbial biomass concentration observed in this study was much lower than those in some results reported before [45]. This phenomenon can also be found in the experiment conducted by Lawrence and McCarty [46], who stated that for non-soluble substrates, the microbial biomass concentration could evidently vary based on VSS.

## 4. Conclusions

In this study, the AD system generally ran stably, and its efficiency improved obviously when the OLR was below 11.5 g VS/(L·d); however, when the OLR reached 11.5 g VS/(L·d), the experiment was inhibited and eventually failed because of the accumulation of TAN. The TS, VS, and COD removal efficiencies reached their maximum values when the TAN concentration was approximately 1600 mg/L. The COD removal efficiency showed that the total organic degradation was quite efficient, especially when the TAN concentration was under 800 mg/L. The microbial biomass balance model revealed that excessively high VS concentration (in Stage 5) led to a low microbial biomass concentration, which may be the main reason why the AD finally failed in this stage. This study may provide a theoretical reference for the optimization of AD performance with nitrogen-rich materials as a substrate by gradually increasing its OLRs.

## Figures and Tables

**Table 1:** Physiochemical characters of the raw chicken manure and inoculum sludge.

Parameters	TS (%)	VS (%)	pH	Alkalinity (mg/L)	TAN (mg/Kg)	TCOD (mg/Kg)
Chicken manure	33.2 ± 0.2	25.6 ± 0.2	7.71 ± 0.2	6270 ± 24.5	2240 ± 11.4	321,800 ± 9700
Inoculum sludge	1.18 ± 0.1	0.74 ± 0.1	7.15 ± 0.1	2151 ± 20.4	236 ± 4.1	3250 ± 200

Note: All relative values were calculated based on wet weight. TS, total solid content; VS, volatile solid content; TAN, total ammonia nitrogen content; TCOD, total chemical oxygen demand.

**Table 2:** Characteristics of the digestate inside the CSTR at each stage.

Stage	OLR (g VS/(L·d))	TCOD (mg/L)	TAN (mg/L)	Alkalinity (mg/L)	pH
1	3.5 ± 0.1	21865 ± 62.9	242 ± 17.6	2360 ± 19.7	6.6 ± 0.2
2	5.5 ± 0.1	33848 ± 136.2	606 ± 38.7	3110 ± 24.8	6.8 ± 0.1
3	8.0 ± 0.1	44000 ± 192.6	1287 ± 96.4	4850 ± 17.7	7.1 ± 0.2
4	11.5 ± 0.1	53183 ± 246.5	1244 ± 137.6	5901 ± 39.4	7.4 ± 0.2
5	15.0 ± 0.1	64702 ± 289.0	1553 ± 176.2	6541 ± 56.2	7.6 ± 0.2

Note: OLR, organic loading rate.

**Table 3:** Comparison of microbial biomass yield values in different OLRs for AD.

OLRs	Substrate	Microbial Biomass Yield (Y)	References
0.3~4.3g/(L·d) *	Pig manure	0.016	[38]
0.3~4.3g/(L·d) *	Pig manure	0.065	[38]
1.0~5.5g/(L·d) **	Food waste	0.051 ****	[40]
1.0~4.0g/(L·d) **	Food waste	0.023 *****	[40]
3.5~15g/(L·d) **	Chicken manure	0.0479	This study
3.965~17.5g/ (L·d) ***	Landfill leachate	0.0538	[41]
2.0~20.0g/(L·d) ***	Milk permeate	0.1808	[42]

Note: * based on TS; ** based on VS; *** based on TCOD; **** Adding trace elements; ***** Not adding trace elements.
